# Dnm1 Is Required for the Focal Clustering of Fis1 on the Mitochondrial Outer Membrane

**DOI:** 10.17912/micropub.biology.001780

**Published:** 2025-08-19

**Authors:** Miyu Sasahara, Shunsuke Matsumoto, Yasushi Tamura

**Affiliations:** 1 Graduate School of Science and Engineering, Yamagata University; 2 Department of Bioscience and Biotechnology, Graduate School of Bioresource and Bioenvironmental Sciences, Kyushu University; 3 Faculty of Science, Yamagata University

## Abstract

In yeast, mitochondrial fission is mediated by the dynamin-like GTPase Dnm1, which is recruited to the mitochondrial outer membrane by its receptor, Fis1. To investigate the spatial distribution of Fis1, we used the CRISPR-Cas9 system to insert the gene fragment encoding mNeonGreen into the *FIS1* gene for its N-terminal tagging. Fluorescence microscopy revealed that mNeonGreen-Fis1 appeared as discrete puncta on mitochondria, in addition to a diffuse signal. Here, we show that the focal clustering of Fis1 is dependent on Dnm1. Our findings provide insight into the spatial organization of membrane proteins, highlighting a mechanism by which a downstream effector can influence the distribution of its upstream receptor.

**Figure 1. Fis1 Clustering on the Mitochondrial Outer Membrane Depends on Dnm1:**
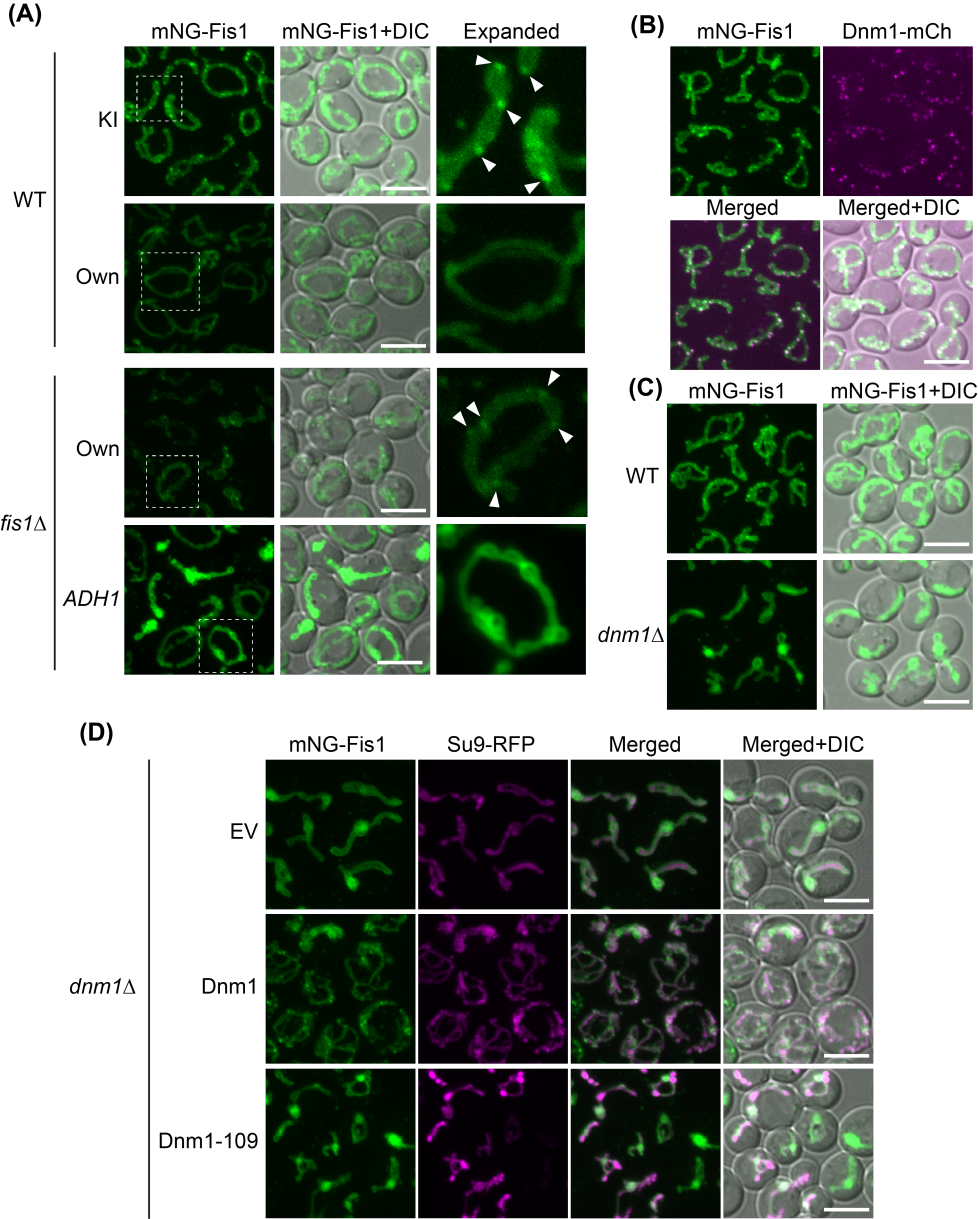
(A) Wild-type cells chromosomally expressing mNeonGreen-Fis1 (mNG-Fis1, KI), wild-type and *fis1* Δ cells expressing mNG-Fis1 from a *CEN* plasmid under the native *FIS1* promoter (Own, pFL270), and *fis1* Δ cells expressing mNG-Fis1 under the constitutive *ADH1* promoter ( *ADH1* , pFL275) were imaged. The region outlined with a dotted line was magnified ("Expanded"). Arrowheads indicate discrete Fis1 puncta. (B) Wild-type cells chromosomally expressing mNG-Fis1 and Dnm1-mCherry (Dnm1-mC). (C) Wild-type and *dnm1* Δ cells chromosomally expressing mNG-Fis1. (D) *dnm1* Δ cells chromosomally expressing mNG-Fis1 and harboring the mitochondrial marker plasmid pFL8 (Su9-RFP), along with either Empty vector (EV), pYC42 (Dnm1), or pYC41 (Dnm1-109). All images were acquired using confocal microscopy. Maximum projection images are shown. Scale bars, 5 µm.

## Description

Determining the subcellular localization of proteins is essential for understanding their functions. To visualize protein localization, fluorescent tags are often fused to the protein and ectopically expressed in cells. However, ectopic expression can result in non-physiological protein levels, which may cause mislocalization due to overexpression or hinder detection due to insufficient expression. To avoid these issues, fluorescent tags are inserted into the genomic locus of the gene of interest, enabling visualization of protein localization under native expression conditions.

In yeast, C-terminal tagging through genomic integration is a widely used and technically straightforward method for studying protein localization (Lequin et al., 2003). However, C-terminal tagging may disrupt proper intracellular targeting, for example in tail-anchored membrane proteins where the C-terminus transmembrane domain plays a critical role in localization (Yofe et al., 2016). Similarly, N-terminal tagging can interfere with organelle-targeting signals at the N-terminus (Kunze and Berger, 2015) and often requires replacing the native promoter with a commonly used constitutive promoter, making it difficult to maintain physiological expression levels.

To overcome these limitations, a precise genome editing technique such as the CRISPR-Cas9 system is a powerful tool, enabling targeted integration of genetic elements into the genome (Okada et al., 2021). This approach allows for the accurate insertion of fluorescent tags while preserving native gene regulation, thereby minimizing artifacts associated with overexpression or mistargeting.

In this study, we used the CRISPR-Cas9 system to insert the gene fragment encoding mNeonGreen immediately upstream of the start codon of the endogenous *FIS1* gene and examined Fis1 localization. Fis1 is a tail-anchored protein of the mitochondrial outer membrane that functions as a receptor for the dynamin-like GTPase Dnm1, which plays a critical role in mitochondrial fission (Mozdy et al., 2000; Tieu and Nunnari, 2000). Fluorescence microscopy revealed that Fis1 showed a diffuse mitochondrial distribution while also accumulating at distinct foci (A). These puncta were not observed when mNeonGreen-Fis1 was expressed from a *CEN* -plasmid under the control of its own promoter in wild-type cells, likely due to excess Fis1 masking its localization (A). Supporting this notion, discrete puncta were detected when mNeonGreen-Fis1 was expressed from the same *CEN* -plasmid in *fis1* Δ cells, although the signal intensity of mNeonGreen-Fis1 was lower than that in cells with genomic expression of mNeonGreen-Fis1 (A). These results indicate that expression level influences Fis1 localization pattern. Further supporting this idea, when mNeonGreen-Fis1 was expressed from a *CEN-* plasmid under the strong *ADH1* promoter, no punctate signals were detected in wild-type and Fis1-lacking cells (A).

We next examined whether Dnm1 colocalizes with the punctate Fis1 signals. Confocal microscopy of endogenously expressed mNeonGreen-Fis1 and Dnm1-mCherry revealed that Dnm1 colocalized with all Fis1 puncta (B). This observation raised two possibilities: either Dnm1 oligomerization promotes Fis1 clustering, or Dnm1 is recruited to sites where Fis1 has already clustered. To distinguish these two possibilities, we analyzed the localization of mNeonGreen-Fis1 in *dnm1* Δ cells. In the absence of Dnm1, punctate Fis1 signals were not detected, indicating that Fis1 clustering depends on Dnm1 (C). To further assess whether Dnm1 oligomerization is specifically required for Fis1 clustering, we expressed an oligomerization-deficient mutant, Dnm1-109, in *dnm1* Δ cells (Cerveny et al., 2007). Under these conditions, Fis1 puncta were not restored, supporting the conclusion that Dnm1 oligomerization is essential for the focal clustering of Fis1 on mitochondria (D). These results highlight a mechanism by which Dnm1, a downstream effector, influences the spatial distribution of the membrane-anchored receptor Fis1, thereby advancing our understanding of membrane protein organization.

## Methods

**Yeast strains**

Saccharomyces cerevisiae strain W303-1A ( *MATa ade2-1 can1-100 his3-11,15 leu2-3,112 trp1-1 ura3-1 rad5-535* ) was used as the background strain. To introduce an N-terminal mNeonGreen tag to Fis1, the CRISPR-Cas9 system was used as previously described (Okada et al., 2021). Briefly, we first selected guide RNA target sequences around the start codons of *FIS1* using CRISPRdirect (Naito et al., 2015). After hybridizing the pair of oligonucleotides (SM112 and SM113) containing the target sequences, they were introduced into the Cas9 expression plasmid 16-15 (Okada et al., 2021) with Golden Gate Assembly Kit (NEB). The resulting plasmids were introduced into yeast cells along with donor DNA fragments encoding mNeonGreen followed by a HRV 3C protease cleavage site, flanked by 50 bp homology arms corresponding to upstream and downstream of the third codon after the start codon of *FIS1* . The donor DNA fragments are PCR-amplified from the pSMQ175 plasmid (Matsumoto et al., 2025) using primers SM114 and SM115. The resulting transformants were cultured in SCGal-Ura medium, and the integration of the desired DNA fragments was confirmed by PCR using genomic DNA as a template using primers SM460/SM461. Finally, the Cas9 expression plasmid was eliminated by culturing the cells in SCD+FOA medium.

To generate yeast strains expressing chromosomal Dnm1-mCherry, a DNA fragment encoding mCherry-KanMX6 with ~50 bp homology arms flanking the DNM1 stop codon was PCR-amplified from pFA6a-mCherry-KanMX6 using primers YU82 and YU83. For DNM1 disruption, a KanMX4 cassette with ~400 bp homology arms corresponding to the promoter and terminator regions was amplified from genomic DNA of fzo1Δ dnm1Δ cells (Kakimoto-Takeda et al., 2022) using primers YU86 and YU89. For FIS1 disruption, a KanMX4 fragment with ~50 bp homology arms targeting the FIS1 promoter and terminator was amplified from pBS-KanMX4 using primers YU1116 and YU1117. PCR products were transformed into mNeonGreen-Fis1-expressing or wild-type cells, and G418-resistant colonies were selected. Correct integration was verified by PCR using primers YU88/YU89 for *DNM1-mCherry,* YU86/YU89 for *dnm1Δ* , and YU1118/YU1119 for *fis1Δ* .

**Growth conditions**

Yeast cells were cultured in YPD (1% (w/v) yeast extract, 2% (w/v) polypeptone, and 2% glucose), SCD (0.67% (w/v) yeast nitrogen base without amino acids, 0.5% (w/v) casamino acids, and 2% (w/v) glucose) or SCGal (0.67% (w/v) yeast nitrogen base without amino acids, 0.5% (w/v) casamino acids, and 2% (w/v) galactose) with appropriate supplements. To eliminate *URA3* -containing plasmids, 5-Fluoroorotic acid was added to SCD medium at a final concentration of 1 µg/ml.

**Fluorescence microscopy**

Yeast cells in the logarithmic growth phase, cultured in YPD or SCD medium, were observed using a model IX83 microscope (Olympus) equipped with a CSU-X1 confocal unit (Yokogawa), a 100× and 1.4 numerical aperture objective lens (UPlanSApo; Olympus), and an sCMOS camera (ORCA-Fusion BT, Andor, Hamamatsu photonics). mNeonGreen or mCherry were excited using a 488-nm or 561-nm laser (OBIS; Coherent), respectively. The confocal fluorescent sections were collected every 0.2 µm from the upper to the bottom surface of yeast cells. Image J software (NIH) was used to create maximum projection images.

## Reagents

**Oligo DNAs used in this study**

**Table:** 

YU82	AATCACTCGGAGTTTATAAAAAGGCTGCAACCCTTATTAGTAATATTCTGCGGATCCCCGGGTTAATTAA
YU83	ATCACGCCCGCAATGTTGAAGTAAGATCAAAAATGAGATGAATTATGCAAGAATTCGAGCTCGTTTAAAC
YU86	CAGCCGGTTCCTGCAAGCAACATCAACACGTTGGC
YU88	AGATGACCTTGAAAACGCTGAACCTCCACTGACCG
YU89	CTAGAAATTTCGTTCATCTAGTTAAAACTATAATC
YU1116	CATAGAAGCACAGATCAGAGCACAGCCATACAACATAAGTGTTGTAAAACGACGGCCAGT
YU1117	TCTTATGTATGTACGTATGTGCTGATTTTTTATGTGCTTGCACAGGAAACAGCTATGACC
YU1118	GTACTACCTTTTCGGGTTGAGTTCG
YU1119	GAAAATTAGCAGCCAAGAACGAGGGC
YU6849	AATTGCGGCCGCCAGTTCAAATAACATGTGTC
YU6850	NNNGGATCCAGGTGTCGTTGCTGCGACA
YU6959	AATTGCGGCCGCATGACCAAAGTCTCTAAGGG
YU6960	NNNGGATCCTTACCTTCTCTTGTTTCTTA
SM112	GGAGCCAACTCTGATGAGTCCGTGAGGACGAAACGAGTAAGCTCGTCAGTTGGCCAAAAATCTACTT
SM113	AAACAAGTAGATTTTTGGCCAACTGACGAGCTTACTCGTTTCGTCCTCACGGACTCATCAGAGTTGG
SM114	ACATAGAAGCACAGATCAGAGCACAGCCATACAACATAAGTATGACCAAAGTCTCTAAGGGTGAAGAAGA
SM115	TGCGGATAGAGTGGTTCGTATGCGTCCTTAAGAGTTGGCCAAAAATCTACGGGTCCCTGAAAGAGGACTT
SM460	CTGTACGCACCAATGTTATCTAC
SM461	GTGCGATTCATTCTTATGTATG

**Plasmids used in this study.**

**Table:** 

Plasmid #	Plasmid Name	Ref.
pYU21	pBS-KanMX4	(Sakaue et al., 2019)
pYU36	pFA6a-mCherry-KanMX6	(Kakimoto et al., 2018)
pYC41	pRS315-Dnm1-109-HA	(Cerveny et al., 2007)
pYC42	pRS316- Dnm1-HA	(Cerveny et al., 2007)
pFL8	pRS316-ADH1p-Su9-RFP	(Tamura et al., 2012)
pFL270	pRS316-Own-mNeonGreen-Fis1	This study
pFL275	pRS316-ADH1-mNeonGreen-Fis1	This study
pSMQ175	pRS304-DDI2pro-3xFLAG-mNeonGreen-HRV3C-PEX15-CYC1ter	(Matsumoto et al., 2025)

pFL270 and pFL275 were constructed as follows. DNA fragments containing either *mNeonGreen-FIS1* with its native *FIS1* promoter and terminator, or the coding region from the start to stop codon of *mNeonGreen-FIS1* , were amplified by PCR using genomic DNA from the mNeonGreen-Fis1-expressing cells as a template and primers YU6849/YU6850 or YU6959/YU6960. The PCR products were cloned into either the NotI/BamHI sites of pRS316 or a pRS316 vector pre-cloned with the *ADH1* promoter and *CYC1* terminator, yielding pFL270 and pFL275, respectively.
